# The relationship between stone-free and patient position in retrograde intrarenal surgery: a randomized prospective study

**DOI:** 10.1007/s00345-024-05013-1

**Published:** 2024-05-09

**Authors:** Cengiz Çanakcı, Erdinç Dinçer, Utku Can, Alper Coşkun, Bilal Kaan Otbasan, Orkunt Özkaptan

**Affiliations:** Department of Urology, Health Sciencies University, Kartal Dr. Lutfi Kirdar City Hospital, D100 Güney Yanyol Cevizli Kartal, Istanbul, Turkey

**Keywords:** Retrograde intrarenal surgery, Patient position, Kidney stone, Stone-free

## Abstract

**Purpose:**

Residual fragments not removed with urinary stone surgery may become symptomatic. In this context, this study was carried out to investigate the effect of performing retrograde intrarenal surgery, which is conventionally performed in the lithotomy position, in the modified lithotomy position (Trend-side) on stone-free rates following the surgery.

**Methods:**

This prospective study consisted of 100 patients with a single kidney stone smaller than 2 cm between 2021 and 2023. These patients were randomized into two groups of 50 patients each to be operated on in the conventional lithotomy and Trend-side positions. Variables were compared using independent t test for continuous variables and chi-square test for categorical variables.

**Results:**

There was no significant difference between the lithotomy and Trend-side position groups in terms of preoperative size, density, location of the stone, and hydronephrosis degree. Stone-free rate was 72% (n = 36) in the lithotomy group and 92% (n = 46) in the Trend-side group. Hence, there was a significant difference between the groups in the stone-free rate in favor of the Trend-side group (p = 0.009). Fragmentation time was statistically significantly shorter in the Trend-side group than in the lithotomy group (34 ± 17 min vs. 43 ± 14 min; p = 0.006). There was no significant difference between the groups in postoperative complication rates.

**Conclusion:**

Performing retrograde intrarenal surgery in the Trend-side position shortened the duration of fragmentation compared to the lithotomy position and was associated with higher stone-free rates. In conclusion, the Trend-side position can be safely preferred in patients undergoing retrograde intrarenal surgery due to kidney stones.

## Introduction

With the development in technology, the use of retrograde intrarenal surgery (RIRS), which is a less invasive surgical technique with lower complication rates compared to other techniques, has increased in the case of kidney stones smaller than 2 cm [1, 2]. Infundibulopelvic angle affects the success of surgery in lower calyx stones. Flexible ureterorenoscopes (fURS) have the capacity to bend up to a certain angle. Given the kidney anatomy, reaching the stone can be challenging in the case of lower calyx stones resulting in lower stone free rates [3].

Stone-free rates after RIRS are reported to be between 72 and 88%. The presence of residual fragments may cause symptoms requiring intervention in the future [4]. Residual fragments are mostly seen in the lower calyx. The steep infundibulopelvic angle and long and narrow infundibulum may obstruct the passage of stones and may not allow the ureterorenoscopy to reach the lower calyx. Laser fibers reduce the deflection of the ureterorenoscope and make it difficult to get to the stone, which may prolong the operation time and increase the risk of damage to the device [5].

**Fig. 1:**
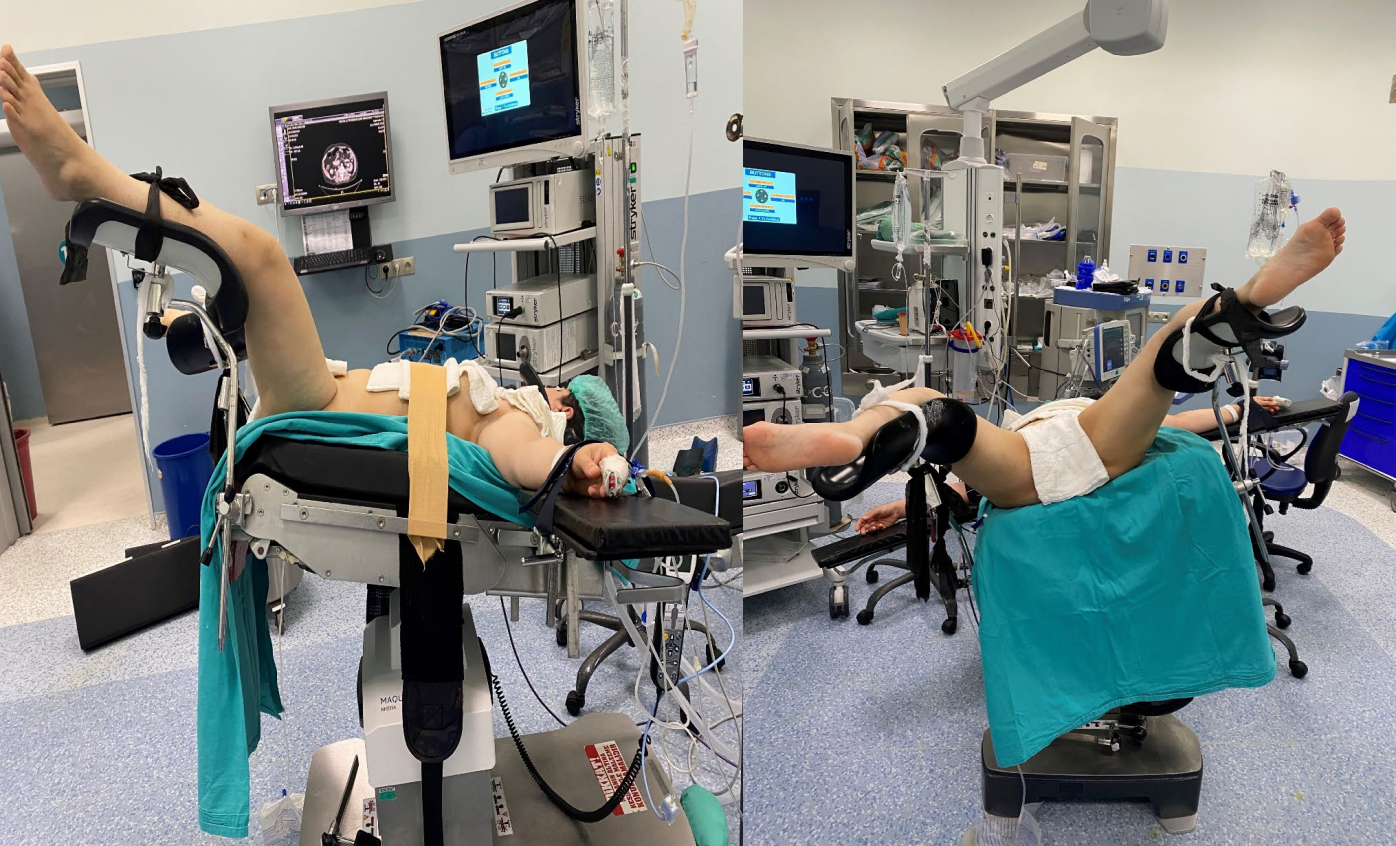
Trend-side position

RIRS is performed in the lithotomy position as standard. The problems experienced in reaching the stone in cases of migration in the lower calyx created the need for studies to reduce stone migration. The position of the patient during surgery may affect the stone migration. There are studies showing that stone-free rates can be increased by changing patients' positioning during surgery [4, 6]. An extensive literature review revealed only one study on the effect of changing patients' operative positioning from the conventional position to another on the success of RIRS. However there is no study that investigated stone free rates and outcomes in a 20 degree and Trend-side position. In the Trend-side position, the patients are turned sideways in a 20-degreeTrendelenburg position while the surgical side faces 20 degrees upwards (Fig. 1).

Performing RIRS in the Trend-side position may increase the migration of stone fragments to the renal pelvis and upper calyx during operation. Afterwards these fragments may most likely to migrate down the ureter rather than back into the lower pole postoperatively. In light of the above, we aimed to evaluate the efficacy of performing RIRS in the Trend-side position compared to the lithotomy position in terms of stone-free rates and complications.

## Material and methods

This study was designed as a prospective randomized study. The study protocol was approved by the local ethics committee (Decision No: 514/194/6, Decision Date: 27.01.2021). The stone-free ratio in the literature on RIRS was utilised to calculate the sample size. Given the stone-free rate of 75% in the lithotomy position, as supported by literature, we considered a 15% difference to be of clinical significance. Based on this calculation, it concluded that a minimum sample size of 49 patients per group would ensure a statistical power of above 80%. The population of this prospective study consisted of 117 patients with a single kidney stone < 2 cm diagnosed between January 2021 and July 2023. Patients with cardiac problems, congenital kidney anomalies, grade 4 hydronephrosis, and patients in whom an access sheath could not be placed were excluded from the study. In the end, the study sample consisted of 100 patients. A simple 1:1 randomization into two groups of 50 patients each to be operated on in the conventional lithotomy and Trend-side positions was performed. Demographic and clinical data of the patients, such as age, gender, body mass index (BMI), whether the kidney stone was in the left or right kidney, and whether a double J (DJ) stent was inserted preoperatively, were recorded. All patients underwent a low-dose computerized tomography (CT) preoperatively. The diameter of the largest stone, stone volume, stone density, and hydronephrosis degree were measured on CT. Stone volume was calculated with the following formula: V = 0.523xAxBxC [7]. The patients randomized into the lithotomy group were operated on in the conventional lithotomy position, whereas the patients randomized into the Trend-side group were operated on in the Trend-side position by turning them sideways in a 20-degreeTrendelenburg position during the surgical side faced 20 degrees upwards. Patients were operated on after a confirmed sterile urine culture. All operations were performed by a single surgeon.

After diagnostic ureteroscopy was performed with an 8/9.8 F ureterorenoscope, a 9.5–11.5 F ureteral access sheath was placed over a guide wire. Patients for whom an access sheath could not be placed forwere excluded from the study. RIRS was performed with a 7.5–8.4 F Hawk brand fURS. Holmium: YAG laser with 272µ fiber was used for stone fragmentation at an energy level of 0.5–1.2 J and pulse rate of 10–15 Hz. Stone fragmentation time was recorded. A 4.8 F DJ stent was placed in all patients at the end of the procedure. Perioperative complications were recorded. Patients were followed up with plain abdominal radiography on the postoperative 1st day. DJ stents were removed at the postoperative 4th week. Stone-free status was evaluated by low-dose thin-section CT in the postoperative 3rd month. Complete stone-free status was assessed on CT. Postoperative complications and rehospitalizations in the postoperative period were recorded.

A diagnostic ureteroscopy was performed, and the access sheath was placed while the patients were in the lithotomy position. The patients to be operated on in the Trend-side position were turned sideways in a 20-degree Trendelenburg position while the surgical side faced 20 degrees upwards. Before the patients were positioned, they were supported from the shoulders and fixed on the operation table from the chest. In this way, it was aimed to direct the stone fragments displaced towards the upper calyx and to increase the stone passage in the postoperative period.

### Statistical analysis

Values were presented as mean ± standard deviation for quantitative variables such as age and BMI. Qualitative variables were presented as number and percentages. Chi square test was used to evaluate categorical variables. The Kolmogorov–Smirnov test was used to analyze the normality of the distribution of variables. The t-test was done to compare continuous variables with normal distribution. The Mann–Whitney U test was used to compare continuous variables with a skewed distribution. The Pearson correlation analysis was performed to evaluate relationships between the variables. All analyses were calculated using the SPSS software version 23.0 (SPSS Inc., Chicago, IL, USA). Statistical tests were two-tailed and a p value of 0.05 was considered significant.

## Results

Of the 117 patients, 112 patients who met the inclusion criteria were included in the study sample. Patients were randomized into two groups. There were 57 patients in the lithotomy group and 55 patients in the Trend-side group. The study excluded 5 lithotomy patients and 4 Trend-side patients due to unplaced access sheaths, and 2 lithotomy patients and 1 Trend-side patient due to non-attendance for follow-up examinations. Eventually, 50 patients in each groups were included in the analysis. There was no significant difference between the groups in terms of age (p = 0.225), gender (p = 0.688), BMI (p = 0.169), and whether the kidney stone was in the left or right kidney (p = 0.198) (Table 1). No significant difference was observed between the two groups concerning the location of the stones (p = 0.716). Renal pelvis stones, which were detected in 24 (48%) patients in the lithotomy group and 22 (44%) patients in the Trend-side group, out of 50 patients in each group, were the most common type of kidney stone in both groups. The stone diameter was 13.26 ± 3.25 mm in the lithotomy group and 13.66 ± 3.67 mm in the Trend-side group. Accordingly, there was no significant difference between the two groups in terms of stone diameter (p = 0.566). The stone volume was 783 ± 444 mm^3^ in the lithotomy group and 987 ± 736 mm^3^ in the Trend-side group. There was no significant difference between the two groups regarding stone volume (p = 0.097). Similarly, there was no significant difference between the two groups in terms of stone density (p = 0,870), hydronephrosis degree (p = 0,984), and preoperative DJ stenting (p = 0,810).

**Table 1:** Distribution of demographic characteristics and perioperative outcome

	Lithotomy Group, *n* (%)	Trend-side group, *n* (%)	*p* value
Age (years), mean ± sd	49.58 ± 13.42	46.22 ± 14.11	0.225
Gender (f/m), *n*	22/28	24/26	0.688
BMI (kg/m^2^), mean ± sd	27.20 ± 2.35	27.94 ± 2.96	0.169
Laterality, *n* (%) Right Left	13(26) 37(74)	19(38) 31 (62)	0.198
Stone location, *n* (%) Upper pole Interpolar Renal pelvis Lower pole Ureteropelvic junction	3 (6) 11 (22) 24 (48) 7 (14) 5 (10)	5 (10) 13 (26) 22 (44) 8 (16) 2 (4)	0.716
Stone size (mm), mean ± sd	13.26 ± 3.25	13.66 ± 3.67	0.566
Stone volume(mm^3^), mean ± sd	783 ± 444	987 ± 736	0.097
Hydronephrosis grade, *n* (%)	Grade 0: 20(40) Grade 1:23(46) Grade 2:7(14) Grade 3:0(0)	Grade 0: 17(34) Grade 1:24(48) Grade 2:7(14) Grade 3:2(4)	0.984
Preoperative DJ stent, *n*	38/12	40/10	0.81
Hounsfield Unit, mean ± sd	748 ± 237	740 ± 254	0.870
Fragmentation time (min.), mean ± sd	43 ± 14	34 ± 17	0.006*
Stone-free rate, *n* (%)	36/14 (72)	46/4(92)	0.017*
Complication rate, *n* (%)	3/29(9.4)	2/32(5.9)	0.592
Readmission rate, *n* (%)	6/44(12)	4/46(8)	0.741

*p value of 0.05 was considered significant

Considering stone-free status, 36 patients (72%) in the lithotomy group and 46 patients (92%) in the Trend-side group were stone-free at the 3-month postoperative period. Accordingly, there was a statistically significant difference between the groups in stone-free rates (p = 0.017) (Table 1). The CT scans of patients with residual pieces indicated that all remaining stones were located in the lower calyx.

Fragmentation time was statistically significantly shorter in the Trend-side group than in the lithotomy group (34 ± 17 min vs. 43 ± 14 min; p = 0.006). Complications associated with patient position was not determined in either of the groups. In relation to postoperative complications, two patients in the lithotomy group experienced fever, while one patient in the same group experienced hematuria. Further, two patients in the Trend-side group had fever. Patients with complications were treated with appropriate antibiotic therapies. There were six and four patients re-hospitalized in this manner in the lithotomy and Trend-side groups, respectively. There was no significant difference between the two groups in terms of readmission rates (p = 0.741) (Table 1).

## Discussion

The study investigated the effect of a modified positioning for RIRS on stone free rates and complication. Increased stone free rates were achieved in the Trend-side position compared to the standard position with similar complication rates. RIRS has been widely used in stone surgery in recent years and is successfully applied even in stones larger than 2 cm. [8, 9]. Residual fragments may cause recurrent symptomatic stones, recurrent infections, and the need for reoperation. Stone-free rates are reported between 70 and 90% in the literature. The large variations between reported stone-free rates may be attributed to the variations in preoperative stone volumes and the size of clinically insignificant residual fragments. There are studies in which residual stones 2, 3, and 4 mm in size were considered clinically insignificant [10–12]. In this study, only cases where total stone clearance was achieved were classified as stone-free.

Another reason for the inconsistency between the stone-free rates reported in various studies is the differences between the postoperative imaging methods used in these studies. There is still no consensus on the imaging modality to be used after RIRS. Residue control is usually performed with kidney, ureter and bladder (KUB) radiography and ultrasonography. The stone-free rates were comparable with the study by Liaw et al., who assessed stone-free rates in patients undergoing RIRS in a modified position. Nevertheless these authors evaluated freeness control with ultrasonography and KUB radiography. Given that these imaging methods do not always provide objective results, stone-free rates can be reported higher than they actually are. Low-dose non-contrast CT, which provides very important information in urinary system stone disease in terms of both diagnosis and treatment, is also the most successful imaging method in showing residual fragments smaller than 2 mm. In addition the study evaluated stone free rates in the postoperative first month. However previous studies reported that the passage of small fragments continued for up to postoperative 3rd months [12, 13]. In our study, stone-free was evaluated with low-dose thin-section non-contrast CT at the postoperative 3rd month to eliminate bias.

The parameters with the highest prognostic values in predicting stone-free status following RIRS are stone size and location of the stone. Postoperative residual fragments are primarily observed in the lower calyx [12, 14]. A number of studies addressed some maneuvers with the aim to increase stone free rates. One of the investigations involved the partial fragmentation and displacement of lower calyx stones into the upper calyx using a basket catheter. The lower calyx was infused with autologous venous blood via a flexible ureterorenoscope (fURS). In the end, a stone-free rate of 94% was attained [15]. Studies have demonstrated that the relocation of stones to the middle-upper calyxes, particularly from the lower calyx, using laser fibre or basket catheter, has been proven to significantly increase stone-free rates. Schuster et al. compared lower calyx stones < 1 cm that were displaced with those that were not displaced. The results showed that the displacement of lower calyx stones led to a higher stone-free rate of 89% compared to 77% in non-displaced stones [16]. A comparable study demonstrated improved stone clearance rates by displacing lower calyceal stones measuring less than 2 cm (90% vs. 83%). In addition to increasing the stone-free rate, displacing the stones may also reportedly prolong the life of the device as it will reduce the duration of fURS deflection [17, 18]. The modified RIRS position is a simple and easy manuever without the need for additional devices for positioning. It is more cost-effective compared to the aforementioned studies that need a basket catheter and other equipment.

There are also studies that investigated the effect of patient position on intrarenal movement of the stone and stone free rates. Bercowsky et al. measured the infundibulopelvic angle at the supine and prone 20º and 45º positions on intravenous urography and determined that positioning the patient 20º upside down in the prone position provided the most appropriate infundibulopelvic angle, facilitating access to the lower calyx with a fURS [9, 19]. In another study, Pan et al. compared the standard lithotomy positioning with the 30º Trendelenburg positioning in patients with proximal ureteral stones. They found that the stone-free rate was higher where there was stone migration. The surgery duration was shorter for patients who were operated on in the Trendelenburg position compared to the standard position [6]. Peng et al. performed RIRS in the lateral position in 21 patients with lower pole stones. They achieved a stone-free rate of 85.7% and did not observe any complication except postoperative fever in only one patient [20]. Similar to our study, Liaw et al. performed RIRS at 15º airplane and 15º Trendelenburg (t-tilt) positions in 2021 and achieved higher stone-free rates both overall (92.1% vs. 76.7%) and only in case of lower calyceal stones (95.6% vs. 68.2%). However, they did not elaborate on why they chose 15º as the positioning angle. These authors did not clarify their hypothesis for choosing a 15 degree t-tilt position. It is known that the LİP angle played the most important role for stone passage. Bercowsky et al. found that the head-down position of 20 degrees was the most suitable LİP for attaining optimal stone passage [4, 19].

There is no consensus on the treatment of asymptomatic kidney stones. The surgical success rate, particularly for asymptomatic lower calyceal stones, is comparatively lower than that for stones located in other areas [21, 22]. Given the high prevalence of kidney stones in our region, patients with lower calyceal stones are typically advised to undergo surveillance. That is the explanation for why we had a reduced number of patients with smaller stones in our research. Extending the surgical procedure increases the risk of infectious complications and injuries. In RIRS, the size of the stone, its location, the number of stones, kidney anatomy, the use of an access sheath, the presence of a preoperative DJ stent, and surgeon's experience affect the duration of the operation. In Sorokin and Ark's study, it was demonstrated that lower pole stones increased the duration of the operation [23]. Our results indicated that the fragmentation time in the Trend-side position was reduced compared to the standard position. However, there was no difference in the rate of complications. A prospective study investigating the outcome of lower calyx stones operated on in the trend-side position would be beneficial.

Another limitation that should be mentioned is the fact that the single surgeon performing all these cases was not blinded to the position. This might affect the outcomes. A multicentric study with multiple surgeons might decrease the risk of bias. Although the sample size was statistically sufficient, the relatively low number of our patients may be deemed a limitation. In fact, the stone-free rates could not be evaluated according to stone location due to the low number of patients. Intent–to–treat analysis is the recommended approach for randomized clinical trials however we have excluded two patients due to cardiac problems and three patients with kidney anomalies were also excluded from the study. This should be mentioned as another weakness of the manuscript.

## Conclusion

Performing RIRS in the Trend-side position shortened the duration of fragmentation compared to the lithotomy position and resulted in a higher number of stone-free patients. Hence, patients who were operated on in the Trend-side position will likely have less risk for symptoms such as recurring kidney stones in the future. In conclusion, the Trend-side position can be safely preferred in patients undergoing RIRS due to kidney stones.
